# BPI overexpression suppresses Treg differentiation and induces exosome-mediated inflammation in systemic lupus erythematosus

**DOI:** 10.7150/thno.63743

**Published:** 2021-10-25

**Authors:** Huai-Chia Chuang, Ming-Han Chen, Yi-Ming Chen, Huang-Yu Yang, Yi-Ru Ciou, Chia-Hsin Hsueh, Ching-Yi Tsai, Tse-Hua Tan

**Affiliations:** 1 Immunology Research Center, National Health Research Institutes, Zhunan, Taiwan.; 2 Division of Allergy, Immunology, and Rheumatology, Taipei Veterans General Hospital, Taipei, Taiwan.; 3 Division of Allergy, Immunology, and Rheumatology, Taichung Veterans General Hospital, Taichung, Taiwan.; 4 Department of Medicine, Chang Gung Memorial Hospital, Taoyuan, Taiwan.; 5 Department of Pathology & Immunology, Baylor College of Medicine, Houston, Texas, USA.

**Keywords:** BPI, SLE, exosomes, Treg, T cells

## Abstract

**Background:** Serum-derived exosomes are correlated with disease severity of human systemic lupus erythematosus (SLE). The proteins in the T-cell-derived exosomes from SLE patients could contribute to inflammation.

**Methods:** We characterized proteins of T cell-derived exosomes from SLE patients and healthy controls by proteomics. To study the potential pathogenic role of the identified exosomal protein, we generated and characterized T-cell-specific transgenic mice that overexpressed the identified protein in T cells using immunohistochemistry, immunoblotting, and single-cell RNA sequencing.

**Results:** We identified an overexpressed protein, bactericidal/permeability-increasing protein (BPI), in SLE T cells and T-cell-derived exosomes. T-cell-specific BPI transgenic (Lck-BPI Tg) mice showed multi-tissue inflammation with early induction of serum IL-1β levels, as well as serum triglyceride and creatinine levels. Interestingly, exosomes of Lck-BPI Tg T cells stimulated IL-1β expression of wild-type recipient macrophages. Remarkably, adoptive transfer of BPI-containing exosomes increased serum IL-1β and autoantibody levels in recipient mice. The transferred exosomes infiltrated into multiple tissues of recipient mice, resulting in hepatitis, nephritis, and arthritis. ScRNA-seq showed that Lck-BPI Tg T cells displayed a decrease of Treg population, which was concomitant with ZFP36L2 upregulation and Helios downregulation. Furthermore, *in vitro* Treg differentiation was reduced by BPI transgene and enhanced by BPI knockout.

**Conclusions:** BPI is a negative regulator of Treg differentiation. BPI overexpression in T-cell-derived exosomes or peripheral blood T cells may be a biomarker and pathogenic factor for human SLE nephritis, hepatitis, and arthritis.

## Introduction

Systemic lupus erythematosus (SLE) is a chronic and debilitating autoimmune disease. SLE manifests systemic inflammation and tissue damages on multiple organs, including the liver, kidney, and joint 1. To date, effective diagnosis and treatment of SLE are still lacking due to heterogeneous symptoms and limited therapeutics 1, 2. Understanding of SLE pathogenesis will help the identification of novel therapeutic targets for SLE.

T cells play important roles in the pathogenesis of SLE by inducing autoantibody production and inflammatory responses 3-5. SLE patients have increased populations of effector/memory T cells and Th17 cells 3, 6, as well as enhanced Th1/Th2 ratio 7, 8. Th1-secreted IFN-γ and TNF-α stimulate macrophage activation and tissue infiltration, leading to multi-organ damages 7. Th17 cells recruit macrophages and dendritic cells to inflammation sites; Th17 cells also facilitate B-cell activation and autoantibody production 9. In contrast, regulatory T cell (Treg) cells are decreased in SLE patients 10, resulting in hyperactivation of effectors T cells. Thus, imbalance of various T-cell populations contributes to SLE pathogenesis.

Cell-derived exosomes directionally deliver mRNAs, microRNAs, proteins, amino acids, or metabolites to targeted cells/tissues, leading to modulation of cell property or tissue environment 11-14. Moreover, T-cell-derived exosomal microRNAs 14-16 and proteins 17 modulate immune responses The serum exosome number in the peripheral blood of SLE patients is increased compared to healthy controls; the serum exosome number is correlated with the SLE disease severity 18. The exosomes isolated from the sera of SLE patients enhance proinflammatory cytokine production from the impacted peripheral blood mononuclear cells of healthy individuals 18. Furthermore, eosinophil cationic protein (ECP, also named human RNase 3)-containing exosomes are involved in SLE pathogenesis 17. SLE serum exosomes also show increased levels of microRNA-21 and microRNA-155; the two exosomal miRNAs are associated with SLE nephritis 19. In addition, SLE exosomal miRNA-146a promotes senescence of mesenchymal stem cells 20; however, miRNA-146a levels are decreased in the serum exosomes of SLE patients compared to those of healthy controls 20.

Bactericidal/permeability-increasing protein (BPI) is a bactericidal protein against gram-negative bacteria 21; BPI binds to the lipid A moiety of LPS and neutralizes endotoxin, leading to attenuation of LPS-induced inflammation 21, 22. In addition to regulation of immune responses, BPI is also involved in metabolic pathways. Gene polymorphism in the BPI 3'-UTR is associated with the increase of both BPI levels and insulin sensitivity 23. Plasma BPI levels are correlated with endothelium-dependent vasodilatation and HDL cholesterol production 24. Furthermore, BPI inhibits angiogenesis by inducing endothelial cell apoptosis and impairing endothelial cell migration 25. To date, the roles of BPI in T-cell functions and autoimmune disease pathogenesis remain unknown. In this report, we characterized T-cell-derived exosomes from SLE patients and identified exosomal BPI as a pathogenic factor for SLE.

## Materials and Methods

### Human samples

This study was conducted in accordance with the Helsinki Declaration. A total of 81 individuals, including 41 healthy individuals, 40 SLE patients, were enrolled in this study. 15 SLE patients and 28 healthy controls were referred to the Division of Immunology and Rheumatology at Taipei Veterans General Hospital in northern Taiwan. 19 SLE patients and 13 healthy controls were referred to the Division of Immunology and Rheumatology at Taichung Veterans General Hospital in central Taiwan. Six SLE patients were referred to the Kidney Institute at Chang Gung Memorial Hospital in northern Taiwan. Peripheral blood collection from healthy controls and patients, as well as experiments were approved by the ethical committee of Taipei Veterans General Hospital (2017-06-003BC), Taichung Veterans General Hospital (#SE17193B), and Chang Gung Memorial Hospital (201601850B0). All study participants provided written informed consent before being enrolled in the study.

### Mice

All animal experiments were performed in the AAALAC-accredited animal housing facilities at National Health Research Institutes (NHRI). All mice (in C57BL/6J background) were used according to the protocols and guidelines approved by the Institutional Animal Care and Use Committee of NHRI. All mice used in this study were maintained in temperature-controlled and pathogen-free cages. Mouse inflammatory phenotypes shown in H&E staining images were independently diagnosed by three pathologists according to ISN/RPS lupus nephritis classification, as well as NIH lupus nephritis activity and chronicity scoring system 26, clinical scoring for arthritis 27, and clinical criteria for SLE hepatitis 28.

### Generation of T-cell-specific human BPI transgenic mice and BPI-deficient mice

A full-length human BPI coding sequence plus a Flag-tag coding sequence were placed downstream of the proximal lck promoter, which drives gene expression specifically in T cells 29-31. The Lck-BPI transgenic moue line in C57BL/6J background was generated using pronuclear microinjection by NHRI Transgenic Mouse Core. To generate BPI-deficient mice, embryonic stem cells of BPI-deficient mice were purchased from EUCOMM (#26769). The embryonic stem cell was injected into blastocysts from the mouse line C57BL/6J to generate chimeric mice by the Transgenic Mouse Core of NHRI.

### Reagents and antibodies

Anti-BPI antibody (PA002783) was purchased from CUSABIO; anti-CD9 antibody (ab92726) was purchased from Abcam. ExoSparkler exosome membrane labeling kit-Green was purchased from Dojido Molecular Technologies. Anti-mCD3-PerCP (clone 145-2C11), anti-mCD4-pacific blue (clone RM4-5), anti-mB220-PerCP-Cy5.5 (clone RA3-6B2), and anti-mCD11b-PE (clone M1/70) monoclonal antibodies were purchased from BD Biosciences. Anti-mIL-1β-FITC (clone NJTEN3) monoclonal antibody was purchased from eBioscience. ELISA kits for IL-1β, IL-6, and TNF-α were purchased from eBioscience. Rheumatoid factor, anti-nuclear antibody, and anti-dsDNA antibody ELISA kits were purchased from Alpha Diagnostic International Inc.

### T-cell-derived exosome isolation

The experiments for isolation of T-cell-derived exosomes were performed using approaches as described previously 17. 8 × 10^6^ murine or human T cells were cultured for 96 h in the absence of stimulation in RMPI-1640 medium (2 mL). To remove cell debris, supernatants were centrifuged for 15 min at 13,000 rpm. T-cell-derived extracellular vesicles (mostly exosomes under 200 nm in diameter) were precipitated from supernatants by ExoQuick (System Biosciences). Precipitated T-cell exosomes were resuspended in PBS and then incubated with anti-CD63 or anti-CD9 magnetic beads (System Biosciences) to isolate T-cell-derived CD63^+^ or CD9^+^ exosomes, then eluted with Exosome Elusion Buffer (20 μL, System Biosciences).

### Preparation of bone marrow-derived macrophages (BMDMs)

Bone marrow cells from femora and tibiae of wild-type mice were flushed with cold RPMI-1640 media. Bone marrow cells were cultured in 6-well plates with 2 mL RPMI-1640 containing 10% FBS, 1% penicillin/streptomycin (Life Technologies), and macrophage colony stimulating factor (25 ng/mL) for 7 days. 2 × 10^6^ BMDMs were treated with T-cell-derived exosomes isolated from supernatants of 8 × 10^6^ murine splenic T cells. After exosome treatment, the BMDMs were harvested and subjected to real-time PCR for cytokines.

### Adoptive transfer of T-cell-derived exosomes

The adoptive transfer experiments were performed using approaches as described previously 17. 8 × 10^6^ murine primary T cells from the lymph node and spleen of Lck-BPI Tg and wild-type mice were cultured for 96 h in RMPI-1640 medium (2 mL) in the absence of stimulation. Purified T-cell-derived exosomes (from 12 mL medium) were suspended in PBS (300 μL) and then intravenously injected every 3 days (up to 9-30 days) into 3 recipient mice (100 μL per mouse). For Figure 4A, ExoSparkler exosome membrane labeling kit was used to label exosomes with green fluorescent dye.

### *In situ* proximity ligation assay (PLA)

*In situ* PLA assays were performed using the Duolink *In situ*-Red kit (Sigma-Aldrich) according to the manufacturer's instructions as described previously 32. Briefly, paraffin-embedded sections were incubated with anti-human BPI (#CSB-PA0027PS, CUSABIO) and anti-CD9 (#60232-1, Proteintech) antibodies, followed by species-specific secondary antibodies conjugated with oligonucleotides (PLA probes, Sigma-Aldrich). After ligation and amplification reactions, PLA signals from each pair of PLA probes in close proximity (< 40 nm; between human BPI and CD9) were visualized as individual red dots and analyzed by Leica TCS SP5 II microscope. Red dots represent human BPI-containing CD9^+^ exosomes.

### Single-cell RNA sequencing data analysis

T cells were purified from the lymph node and spleen of Lck-BPI transgenic and wild-type mice. Lck-BPI transgenic or wild-type T cells were processed by BD Rhapsody Single-Cell Analysis System. The scRNA-seq data were analyzed by BD SEQGEQ (BD Biosciences) software, as well as the R package Seurat. Uniform Manifold Approximation and Projection (UMAP) was used to perform dimensionality reduction; individual subsets of variable genes were further subjected to clustering analysis.

### T-cell differentiation and proliferation

*In vitro* T-cell differentiation using murine splenic CD4^+^ T cells were performed as described previously 31. Methods of T-cell purification and T-cell proliferation using CFSE-dye dilution were described in previous publications 31, 33, 34.

### Statistics

*In vivo* experiments were conducted using distinct samples; *in vitro* experiments were performed in at least 3 independent experiments. Statistical analyses were performed by using Excel, SPSS, or BD SEQGEQ. Two groups were compared by two-tailed or one-tailed unpaired Student's t-test, as well as Wilcoxon rank-sum test. P values lower than 0.05 were considered significant.

## Results

### Induction of BPI in T-cell-derived exosomes and T cells of SLE patients

**Figure 1:**
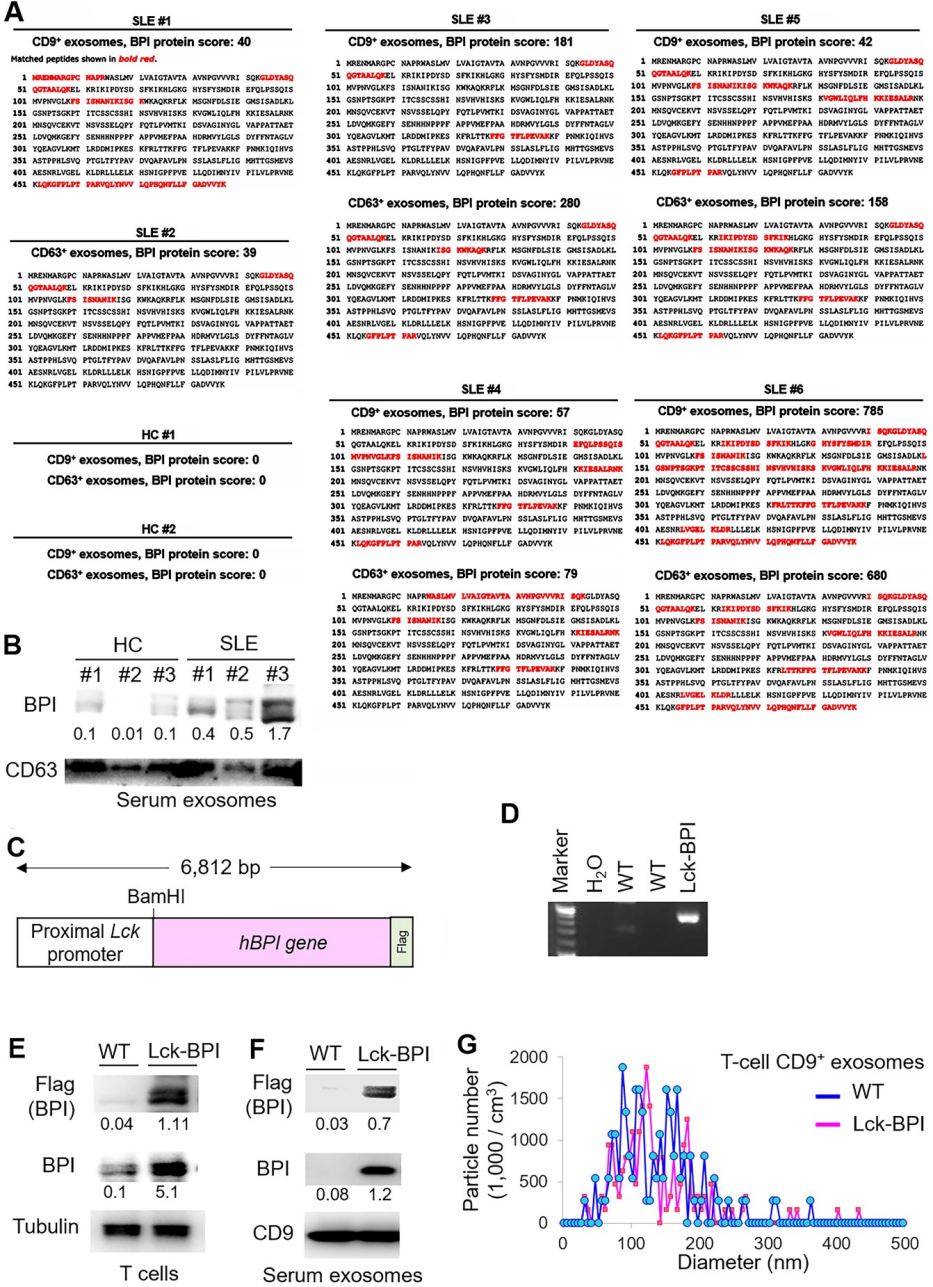
** BPI is enriched in T-cell-derived exosomes of SLE patients.** (**A**) Identification of BPI by mass spectrometry-based protein sequencing of CD9^+^ or CD63^+^ exosomes derived from T cells of SLE patients. The protein score is the sum of the highest ions score of MS/MS search for each distinct sequence. Matched peptides shown in bold red. (**B**) Immunoblotting analysis of BPI and CD63 protein levels in exosomes isolated from 200 µL sera of SLE patients or healthy controls (HC). Relative fold changes were normalized to CD63 levels and are shown at the bottom of the panel. (**C**) Schematic diagram for the construction of the Lck promoter-driven human BPI plus a Flag tag. (**D**) Genotyping of T-cell-specific BPI transgenic (Lck-BPI) mice and wild-type (WT) mice. (**E**) Immunoblotting analysis of transgenic Flag-tagged human BPI protein levels in splenic T cells from Lck-BPI Tg and WT mice. (**F**) Immunoblotting analysis of Flag-tagged BPI and CD9 protein levels in T-cell-derived exosomes from Lck-BPI Tg and WT mice. Relative fold changes (means of three independent experiments) for immunoblotting analyses were normalized to tubulin (E) or CD9 (F) levels and are shown at the bottom of individual panels. (**G**) ZetaView analysis of particle numbers and sizes of extracellular vesicles (EVs) in supernatants from Lck-BPI and WT T cells. EVs were isolated by ExoQuick-TC. Data shown (D, E, F, and G) are representatives of three independent experiments.

To characterize the exosomal proteins of T-cell-derived exosomes from SLE patients, CD9^+^ or CD63^+^exosomes derived from T cells of SLE patients and healthy controls (HCs) were subjected to mass spectrometry-based proteomics (Table 1). The proteomics data showed that the protein bactericidal/permeability-increasing protein (BPI) was detected in CD9^+^ and/or CD63^+^ T-cell-derived exosomes from all 6 SLE patients but not from 2 HCs (Figure 1A). Moreover, immunoblotting analyses confirmed the induction of exosomal BPI in the serum exosomes of SLE patients (Figure 1B), whereas the levels of soluble BPI levels were not significantly increased in the sera of SLE patients (Figure S1A-B). To study whether BPI is overexpressed in SLE T cells, peripheral blood T cells isolated from two SLE patients (#1 and #2) and two HC were also subjected to proteomics; the data showed an induction of BPI in SLE T cells but not in healthy control (HC) T cells (Figure S1C).

### T-cell-specific BPI transgenic mice spontaneously develop inflammatory responses

**Figure 2:**
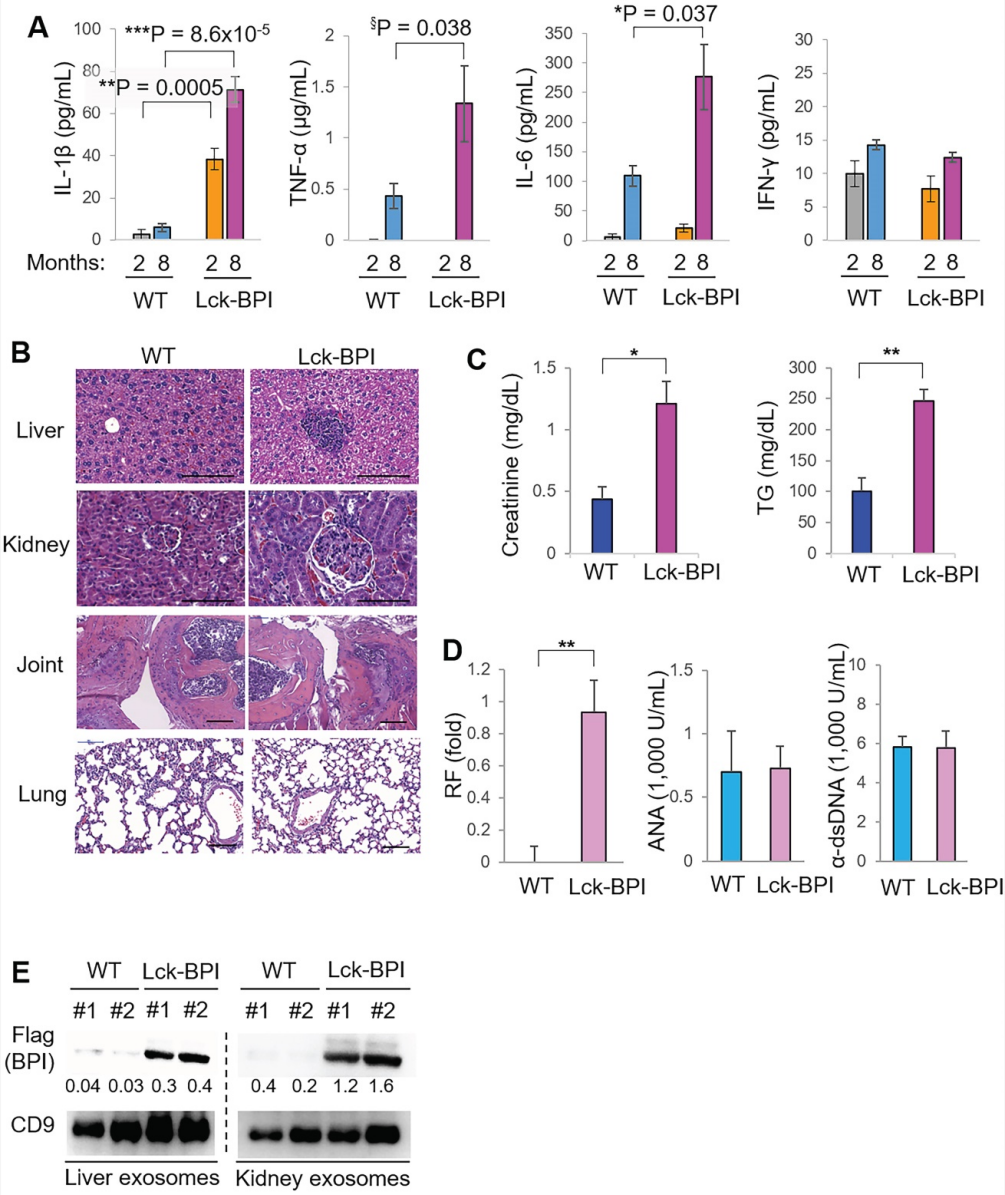
** Lck-BPI transgenic mice spontaneously develop inflammatory phenotypes.** (**A**) ELISA of serum IL-1β, TNF-α, IL-6, and IFN-γ, from 2-month-old mice (WT, n = 4; Lck-BPI, n = 4) or 8-month-old mice (WT, n = 3; Lck- BPI, n = 6). Means ± SEM are shown. *, *P* value < 0.05; **, *P* value < 0.01; ***, *P* value < 0.001 (two-tailed Student's t-test); §, *P* value < 0.05 (one-tailed Student's t-test). (**B**) Hematoxylin and eosin (H&E)-stained sections of the liver, kidney, and joint from 8-month-old Lck-BPI Tg and WT mice. Scale bars, 100 µm. For the liver, the immune cell aggregation regions were presented as the ratio of aggregation area divided by the entire view area. WT, 5.4 ± 7.1 x 10^-5^; Lck-BPI Tg, 1.8 ± 3.6 x 10^-2^. Data shown are mean ± SD of 5 images. For the kidney, the diameters of glomeruli were 60.1 ± 10.7 µm for WT mice and 92.6 ± 10.58 µm for Lck-BPI Tg mice. The mesangial cell numbers were 50.5 ± 12.5/glomerulus for WT mice and 82.5 ± 15.8/glomerulus for Lck-BPI Tg mice. Data shown are mean ± SD of 10 glomeruli. (**C**) Serum creatinine and triglyceride (TG) of 36-week-old wild-type or Lck-BPI transgenic mice were determined using serum chemistry assays. n = 5 per group. (**D**) ELISA of serum anti-nuclear antibody (ANA), rheumatoid factor (RF), and anti-dsDNA antibody in wild-type and Lck-BPI Tg mice. (**E**) Immunoblotting analysis of Flag-tagged BPI and CD9 protein levels in exosomes isolated from the liver (left panel) or kidney (right panel) tissues. Relative fold changes (means of three independent experiments) were normalized to CD9 levels and are shown at the bottom of the Flag panel. Data shown (A-E) are representatives of three independent experiments.

To investigate whether BPI overexpression in T cells plays an important role in the pathogenesis of SLE, we generated and characterized T-cell-specific BPI transgenic (Lck-BPI Tg) mice (Figure 1C-D). The BPI protein was successfully overexpressed in exosomes derived from the T cells and sera of Lck-BPI Tg mice (Figure 1E-F). The sizes of T-cell-derived CD9^+^ or CD63^+^ exosomes of Lck-BPI Tg mice were mostly under 200 nm in diameter (Figure 1G and Figure S2). Five-week-old Lck-BPI Tg mice displayed normal T-cell development in the thymus (Figure S3A-D); interestingly, peripherally derived natural Treg (nTreg) populations of Lck-BPI Tg mice were modestly decreased in the spleen and lymph nodes (Figure S3E-F). To study whether BPI overexpression in T cells induces inflammatory responses in mice, we monitored serum cytokine levels of Lck-BPI Tg mice. Two-month-old Lck-BPI Tg mice spontaneously developed inflammation with increased levels of the proinflammatory cytokine IL-1β, but not TNF-α and IL-6, in the sera compared to those of wild-type mice (Figure 2A). Besides IL-1β, the serum levels of TNF-α and IL-6 were later increased in eight-month-old Lck-BPI Tg mice (Figure 2A). Eight-month-old Lck-BPI Tg mice showed increased infiltrating immune cells in the liver, kidney, and the sublining layer of the joint synovium (Figure 2B). H&E staining of the liver showed immune cell aggregation and hepatocyte spotty necrosis in Lck-BPI Tg mice (Figure 2B and Figure S4). H&E staining of the kidney showed glomerular hypertrophy, mesangial expansion, and glomerular basement membrane thickening in the Lck-BPI Tg mice (Figure 2B and Figure S4). Lck-BPI Tg mice showed manifested class III of lupus glomerulonephritis (nephritis activity score: 4; chronicity score: 1). H&E staining of the joint also showed cartilage destruction and leukocyte infiltration in Lck-BPI Tg mice, indicative of arthritis development (Figure 2B and Figure S4). Serum levels of creatinine and triglyceride (TG) were also significantly increased Lck-BPI Tg mice (Figure 2C), suggesting the development of nephritis and hepatitis, respectively, in Lck-BPI Tg mice. Furthermore, serum levels of the autoantibody rheumatoid factor (RF) were increased in Lck-BPI Tg mice compared to those in wild-type mice (Figure 2D), suggesting the induction of autoimmune responses in Lck-BPI Tg mice. To study whether tissue inflammation is induced by exosomes in Lck-BPI Tg mice, we examined the exosomes from the inflamed tissues. Flag-tagged BPI proteins were detected in the exosomes isolated from the liver and kidney of Lck-BPI Tg mice (Figure 2E). Collectively, T-cell-specific BPI transgenic mice spontaneously develop multi-tissue inflammation with early induction of IL-1β.

### BPI transgenic T-cell-derived exosomes stimulate IL-1β production from macrophages

**Figure 3:**
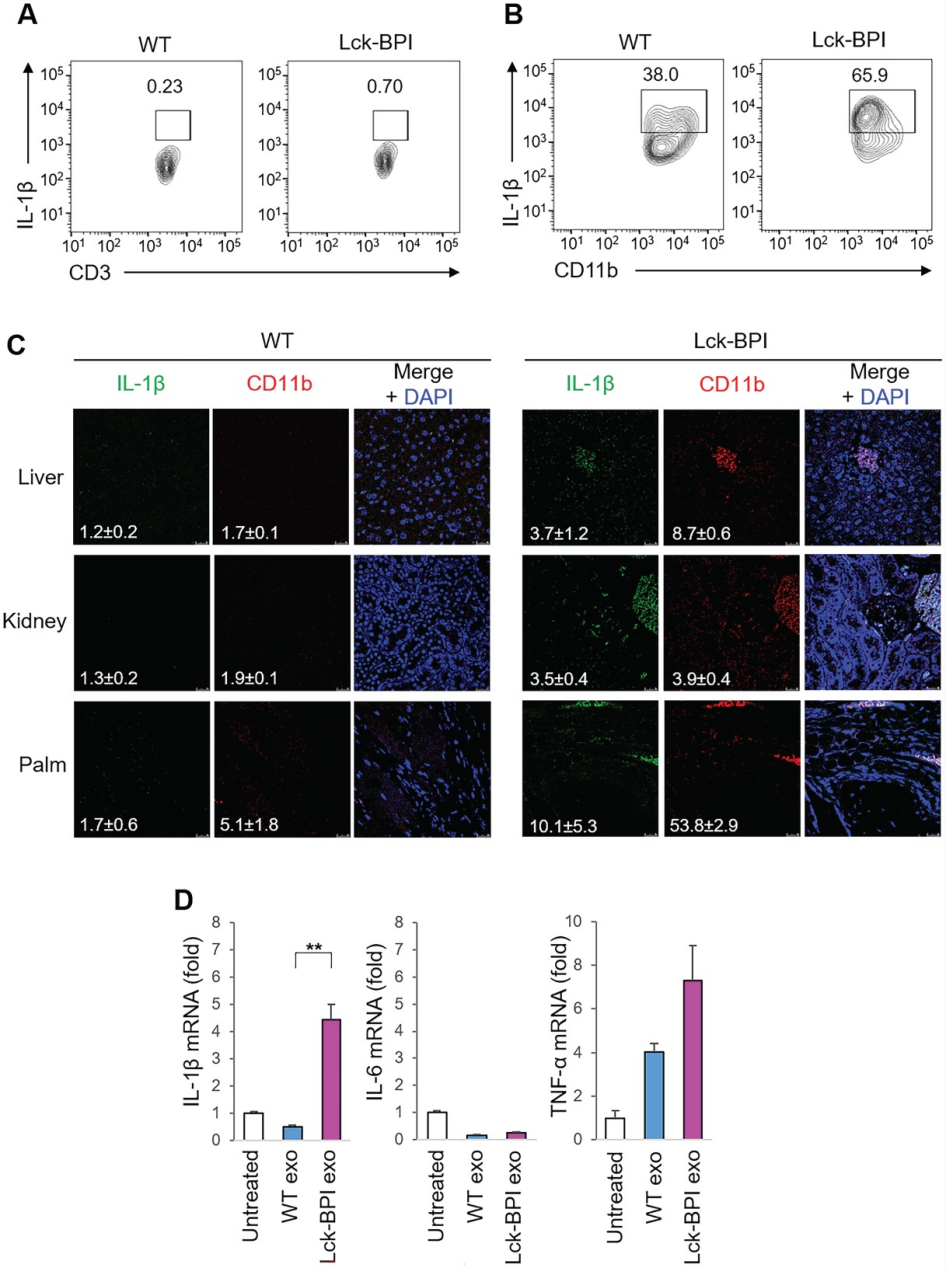
** BPI transgenic T-cell-derived exosomes stimulate IL-β production from macrophages.** (**A** and** B**) Flow cytometry analyses of IL-1β-producing T cells (CD3-gated cells; A) and IL-1β-producing myeloid-lineage cells (CD11b-gated cells; B) from the lymph node of 12-week-old wild-type (WT) or Lck-BPI transgenic mice (**C**) Immunohistochemical staining of FITC-conjugated anti-IL-1β antibody (green) and PE-conjugated anti-CD11b antibody (Red) in the paraffin-embedded sections of the liver, kidney, and palm from wild-type and Lck-BPI Tg mice. Cell nuclei were stained with DAPI (blue). Scale bars, 25 µm. Mean ± SEM of relative fluorescence intensity values (FITC versus DAPI or PE versus DAPI, 10^-6^/um^2^) from 5 images are shown at the bottom of individual panels. (**D**) Real-time PCR of IL-β, IL-6, and TNF-α mRNA levels in wild-type BMDMs stimulated with T-cell-derived exosomes from wild-type or Lck-BPI Tg T cells. Means ± SD are shown. **, *P* value < 0.01 (two-tailed Student's t-test). Data shown (A-D) are representatives of three independent experiments.

IL-β is mainly produced by myeloid-lineage cells instead of T cells; therefore, we studied whether T-cell-derived exosomes from Lck-BPI Tg T cells stimulate IL-1β production from macrophages. As expected, IL-β-producing myeloid-lineage (CD11b^+^) cells, but not IL-β-producing T (CD3^+^) cells, were detected in the lymph node of mice (Figure 3A-B). Consistent with induction of serum IL-β in Lck-BPI Tg mice (Figure 2A), the frequency of IL-β-producing myeloid-lineage (CD11b^+^) cells was increased in the lymph node of Lck-BPI Tg mice compared to those of wild-type mice (Figure 3A). Moreover, IL-β-producing myeloid-lineage (CD11b^+^) cells were detected in the liver, kidney, and palm of Lck-BPI Tg mice (Figure 3C). Interestingly, we found that the exosomes derived from Lck-BPI Tg T cells stimulated IL-1β mRNA levels of wild-type recipient bone marrow-derived macrophages (BMDMs) (Figure 3D). These results suggest that T-cell-derived exosomes stimulate the production of IL-1β, but not IL-6 and TNF-α, from macrophages in Lck-BPI Tg mice.

### BPI transgenic T-cell-derived exosomes induce inflammatory responses in recipient mice

**Figure 4:**
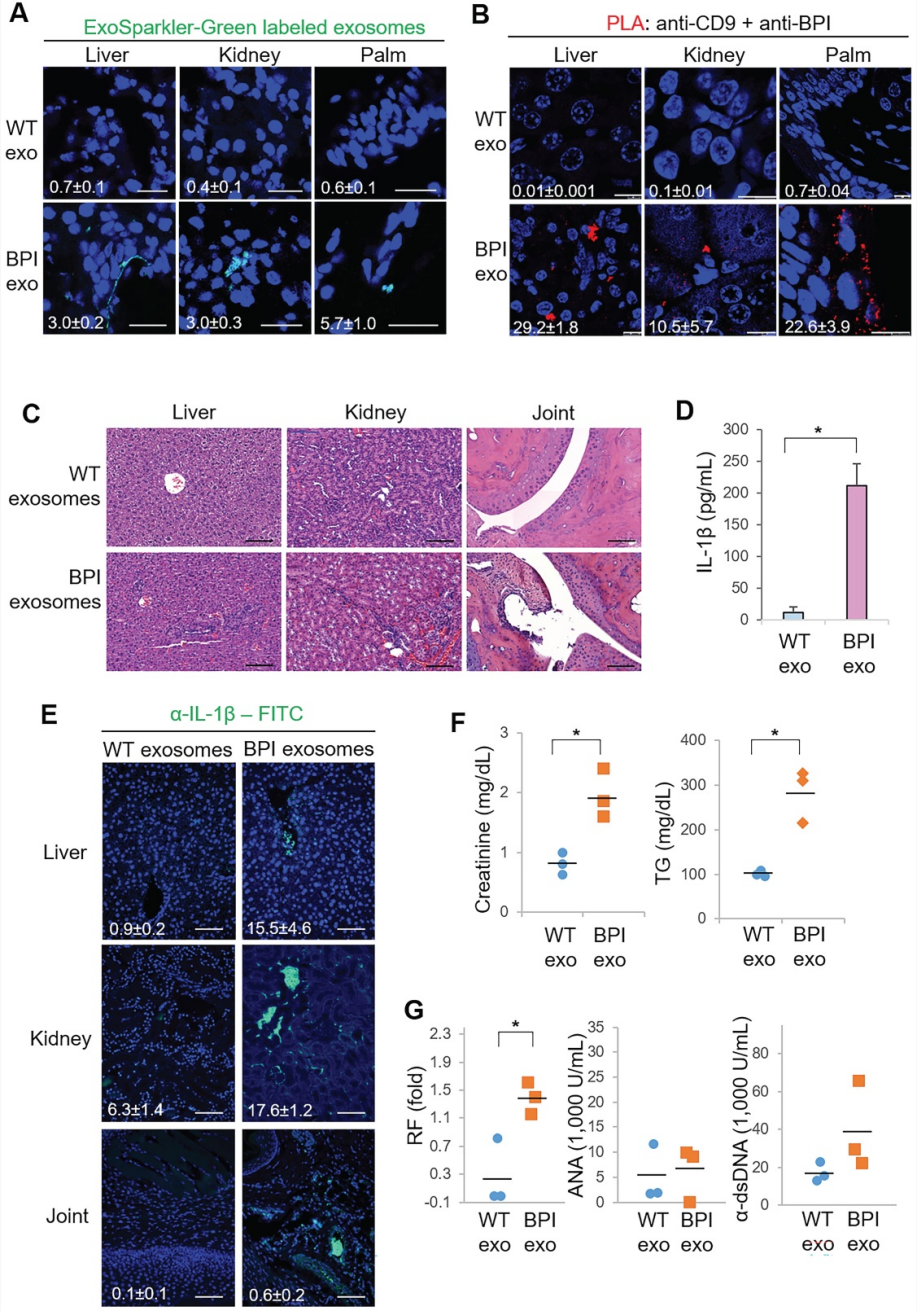
** BPI transgenic T-cell-derived exosomes induce inflammation in recipient mice.** (**A**) Confocal microscopy analysis of fluorescent dye (green)-labeled exosomes in the liver, kidney, and palm of wild-type recipient mice. Exosomes were isolated from supernatants of WT or Lck-BPI Tg T cells by ExoQuick-TC. ExoSparkler-Green labeled exosomes were adoptively transferred into wild-type recipient mice by intravenous injection every 3 days for 9 days. Cell nuclei were stained with DAPI (blue). Original magnification, ×630; scale bars, 25 µm. Mean ± SEM of relative fluorescence intensity values (FITC versus DAPI, 10^-6^/um^2^) from 5 images are shown at the bottom of individual panels. (**B**) *In situ* PLA assays of close proximity (<40 nm) between BPI and CD9 as BPI-containing exosomes in the liver, kidney, and palm of wild-type recipient mice using anti-human BPI antibody plus anti-CD9 antibody. Cell nuclei were stained with DAPI. Original magnification, x630. Scale bars, 10 µm. Mean ± SEM of relative fluorescence intensity values (PLA versus DAPI, 10^-6^/um^2^) from 5 images are shown at the bottom of individual panels. (**C-F**) Exosomes derived from wild-type or Lck-BPI T cells (WT exo or BPI exo) were adoptively transferred into recipient mice by intravenous injection every 3 days for 24 days. n = 3 (biological replicates) per group. (**C**) Hematoxylin and eosin (H&E)-stained sections of the liver, kidney, and joint from recipient mice. Scale bars, 100 µm. For the liver, the immune cell aggregation regions were presented as the ratio of aggregation area divided by the entire view area. WT-exosome recipient mice, 2.5 ± 1.1 x 10^-5^; BPI-exosome recipient mice, 7.3 ± 2.6 x 10^-4^. Data shown are mean ± SD of 5 images. For kidney, the diameters of glomeruli were 55.8 ± 7.3 µm for WT-exosome recipient mice and 81.8 ± 14.7 µm for BPI-exosome recipient mice. The mesangial cell numbers were 41.0 ± 10.6/glomerulus for WT-exosome recipient mice and 70.7 ± 9.5/glomerulus for BPI-exosome recipient mice. Data shown are mean ± SD of 10 glomeruli. (**D**) ELISA of serum IL-1β levels in recipient mice. (**E**) Immunohistochemical staining of FITC-conjugated anti-IL-1β antibody (green) in the paraffin-embedded sections of the liver, kidney, and joint from recipient mice. Cell nuclei were stained with DAPI (blue). Scale bars, 100 µm. Mean± SEM of relative fluorescence intensity values (FITC versus DAPI, 10^-6^/um^2^) from 5 images are shown at the bottom of individual panels. (**F**) Serum creatinine and triglyceride (TG) of wild-type recipient mice were determined using serum chemistry assays. (**G**) ELISA of serum anti-nuclear antibody (ANA), rheumatoid factor (RF), and anti-dsDNA antibody in recipient mice. *, *P* value < 0.05 (two-tailed Student's t-test). Data shown (A-G) are representatives of three independent experiments.

To validate the pathogenic function of BPI-containing exosomes in inflammation, the exosomes isolated from Lck-BPI Tg T cells (hereinafter BPI exosomes) were adoptively transferred into wild-type recipient mice. ExoSparkler-Green labeled BPI exosomes infiltrated into the tissues of the liver, kidney, and palm of wild-type recipient mice after adoptive exosome transfer (Figure 4A). To further confirm the detected fluorescence signals are indeed from the adoptively transferred exosomes but not dissociated dyes from labeled exosomes, we performed the *in situ* proximity ligation assay (PLA) using anti-human BPI antibody plus anti-CD9 to detect close proximity (< 40 nm) between BPI and CD9 as BPI exosomes. The PLA signals were detected in the liver, kidney, and palm of recipient mice adoptively transferred with BPI exosomes but not wild-type exosomes (Figure 4B), supporting that the adoptively transferred BPI exosomes entered into the liver, kidney, and palm of recipient mice. H&E staining results showed induction of infiltrating immune cells in the liver, kidney, and joint of BPI-exosome recipient mice (Figure 4C and Figure S5). Moreover, adoptive transfer of BPI exosomes resulted in induction of IL-1β levels in sera and tissues of the recipient mice (Figure 4D-E). Furthermore, BPI exosomes derived from Lck-BPI T cells enhanced serum levels of creatinine and triglyceride (Figure 4F), as well as the autoantibody rheumatoid factor (Figure 4G) in the recipient mice. These results suggest that the T-cell-derived exosomal protein BPI induces to inflammatory responses.

### BPI overexpression induces ARCN1, ZFP36L2, and S1PR1 in T cells

**Figure 5:**
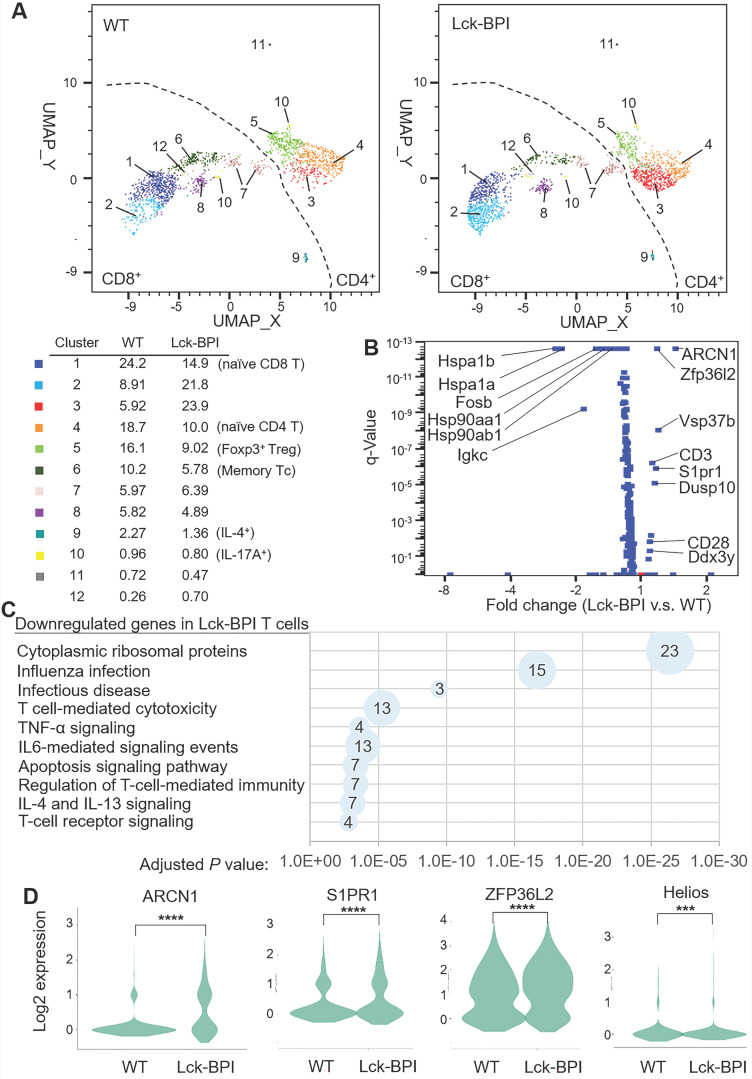
** BPI overexpression in T cells induces ARCN1, ZFP36L2, and S1PR1 expression.** (**A**) Distribution and classification of T cells from wild-type (WT) and Lck-BPI transgenic mice. Data were shown in UMAP. (**B**) The volcano plot shows the selected differentially expressed genes (DEGs) of Lck-BPI Tg T cells vs. WT T cells. (**C**) KEGG pathway enrichment of downregulated genes in whole Lck-BPI transgenic T cells. Pathways belonging to different classifications are listed on the left of the plot. Varied numbers of genes enriched in individual pathways were presented by different diameter sizes and numbers for individual dots. (**D**) Violin plots for the DEGs, ARCN1, S1PR1, ZFP36L2, and Helios, in WT and Lck-BPI T cells. *P* values were determined using Wilcoxon rank-sum test.

To study the underlying mechanism of BPI-induced inflammation, T cells of Lck-BPI Tg mice were isolated and subjected to scRNA-seq. According to different gene signatures, clustering analyses showed 12 distinct clusters in T cells of Lck-BPI and wild-type mice (Figure 5A). Clusters 3, 4, 5, 10, and 11 were identified as CD4^+^ T cells, while Clusters 1, 2, 6, 7, 8, 9, 10, and 12 were identified as CD8^+^ T cells (Figure 5A). Expression levels of 9 genes were significantly upregulated, while 63 genes were downregulated in Lck-BPI Tg T cells (Figure 5B). Kyoto Encyclopedia of Genes and Genomes (KEGG) pathway analyses revealed that these 63 downregulated genes in Lck-BPI Tg T cells belong to apoptosis signaling pathway, IL-4 and IL-13 signaling, and regulation of T-cell-mediated immunity (Figure 5C). Lck-BPI Tg mice showed induction of Cluster 3, which displayed downregulation of genes belonging to downstream signaling in naïve CD8^+^ T cells and infectious disease (Figure S6).

Notably, the most upregulated gene in Lck-BPI Tg T cells was Archain 1 (ARCN1) (Figure 5B-D), also named coatomer delta subunit (COPD) or archain vesicle transport protein 1 35. ARCN1-overexpressing T cells were present in every T-cell clusters of Lck-BPI Tg mice (Figure S7), suggesting that BPI overexpression intrinsically induces ARCN1 levels in all T-cell subsets. Moreover, sphingosine-1-phosphate receptor 1 (S1PR1) (Figure 5B-D) and VPS37b (Figure 5B) were significantly upregulated in Lck-BPI Tg T cells.

**Figure 6:**
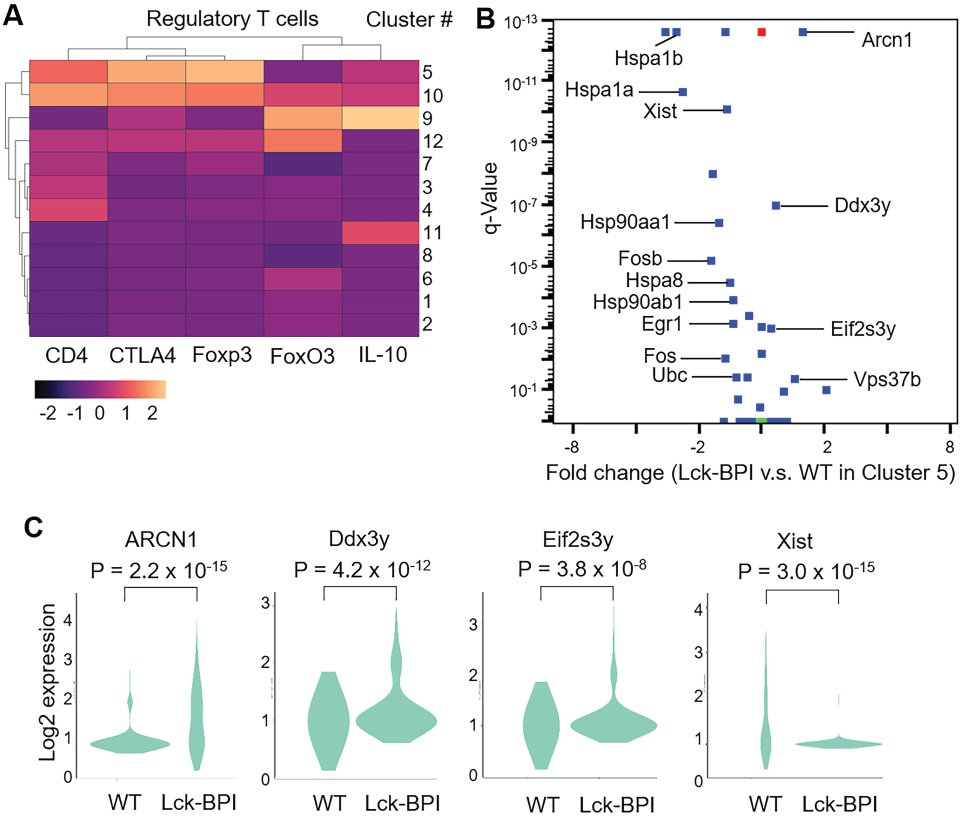
** Treg cell population is decreased in Lck-BPI transgenic mice.** (**A**) Gene-expression heat map of the Treg-related genes in each cluster compared to all other clusters. Genes are represented in columns, and cell clusters in rows. (**B**) The volcano plot shows the selected differentially expressed genes (DEGs) of Lck-BPI Tg T cells vs. WT T cells in Cluster 5. (**C**) Violin plots for the DEGs, ARCN1, Ddx3y, Eif2s3y, and Xist, of WT and Lck-BPI T cells in Cluster 5. *P* values were determined using Wilcoxon rank-sum test.

Lck-BPI Tg T cells also displayed upregulation of ZFP36 ring finger protein-like 2 (ZFP36L2) (Figure 5B-D), which inhibits Treg population and Treg activity by downregulating Helios transcription 36, 37. Consistently, Helios expression was significantly decreased in Lck-BPI Tg T cells (Figure 5D). These results suggest that Treg population may by dysregulated in Lck-BPI transgenic mice. Cluster 5 showed Treg gene signature and CD4 expression (Figure 6A), indicating that Cluster 5 is the conventional Treg population. The data showed decreased Treg population (Cluster 5) in Lck-BPI Tg mice (Figure 5A); these results were consistent with a modestly reduced peripheral nTreg population analyzed by flow cytometry (Figure S3). Furthermore, in the Treg population (Cluster 5), levels of several Treg-downregulated genes such as ARCN1, Ddx3y, and Eif2s3y 38 were increased in Lck-BPI Tg mice compared to those of wild-type mice (Figure 6B-C). In contrast, levels of the Treg-upregulated gene Xist 38 were decreased in Lck-BPI Tg mice (Figure 6B-C). Collectively, the data suggest that BPI overexpression in T cells suppresses Treg population, leading to induction of inflammatory responses.

### BPI negatively regulates iTreg differentiation

**Figure 7:**
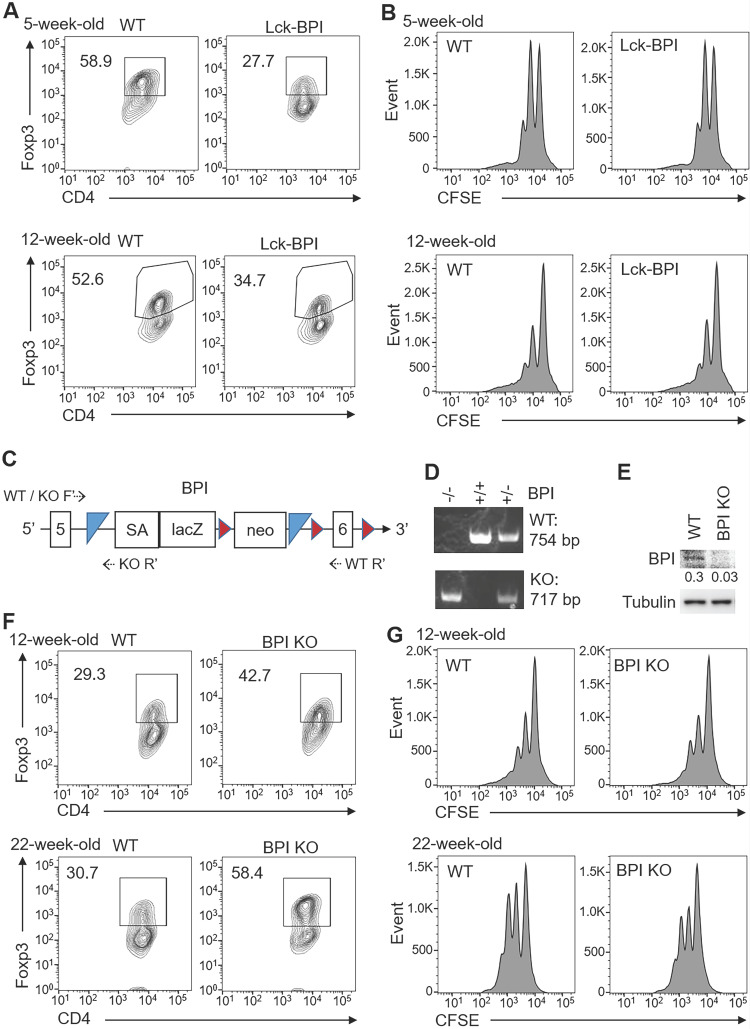
** BPI suppresses Treg differentiation.** (**A**) *In vitro* Treg differentiation of CD4^+^ splenic T cells from 5-week-old or 12-week-old Lck-BPI Tg and WT mice. Foxp3-positive CD4^+^ T cells were analyzed by flow cytometry. (**B**) Suppression of CFSE-labeled CD3^+^ T cells by Treg cells from 5-week-old and 12-week-old Lck-BPI Tg or wild-type mice, presented as CFSE dilution in responding T effector cells cultured with Treg cells at a ratio of 4:1. T cells were stimulated with plate-bound anti-CD3 antibody for 96 h. (**C**) The structure of the gene-trap BPI gene. LacZ, β-galactosidase; Neo, neomycin phosphotransferase genes; SA, splicing acceptor; the box with numbers, the exon of BPI; blue triangle, Flp recombination target (FRT) site; brown triangle, loxp site; dotted arrow, the primers for genotyping PCR. (**D**) PCR analyses of BPI wild-type and mutant allele in the genomic DNA from mouse tails. The PCR products of the higher band (754 bp) indicate wild-type (WT) allele, and the lower band (717 bp) indicates BPI mutant allele. KO, BPI-deficient. (**E**) Immunoblotting analysis of BPI and tubulin protein levels in peripheral blood T cells of wild-type and BPI-deficient mice. Relative fold changes (means of three independent experiments) were normalized to tubulin levels and are shown at the bottom of the BPI panel. WT, wild-type; KO, BPI-deficient. (**F**) *In vitro* Treg differentiation of CD4^+^ T cells from 12-week-old and 22-week-old wild-type (WT) and BPI-deficient (BPI KO) mice. Foxp3-positive CD4^+^ T cells were analyzed by flow cytometry. (**G**) Suppression of CFSE-labeled CD3^+^ T cells by Treg cells isolated from 12-week-old and 22-week-old BPI-deficient (KO) or wild-type mice, presented as CFSE dilution in responding T effector cells cultured with Treg cells at a ratio of 4:1. T cells were stimulated with plate-bound anti-CD3 antibody for 96 h. Data shown (A, B, D, E, F, and G) are representative of at least three independent experiments.

We next studied whether ZFP36L2 overexpression and Helios downregulation in Lck-BPI Tg T cells result in downregulation of induced Treg differentiation. *In vitro* differentiation using primary splenic T cells from 5-week-old or 12-week-old Lck-BPI Tg mice showed that *in vitro* Treg differentiation was reduced by BPI overexpression (Figure 7A), whereas *in vitro* Treg activity of Lck-BPI transgenic Treg was normal under an equal cell number of BPI Tg Treg and wild-type Treg (Figure 7B). *In vitro* Th1 and Th17 differentiation were normal in T cells of 5-week-old Lck-BPI Tg mice, while those were slightly enhanced in T cells of 12-week-old Lck-BPI Tg mice (Figure S8). The results derived from young mice suggest that BPI overexpression in T cells only inhibits differentiation of Treg but not Th1 and Th17. To further confirm the negative role of BPI in Treg differentiation, we generated BPI-deficient mice using the gene-trapped approach (Figure 7C, 7D). BPI ablation in peripheral blood T cells of BPI-deficient mice was confirmed by immunoblotting analysis (Figure 7E). In contrast to BPI overexpression, Treg differentiation was enhanced by BPI deficiency under a suboptimal Treg differentiation condition (Figure 7F), while Treg activity of Lck-BPI Tg mice was similar to that of wild-type mice (Figure 7G). Collectively, these results suggest that Lck-BPI Tg mice display autoinflammation partly due to decreased Treg differentiation.

## Discussion

A key finding of this study was the identification of one novel T-cell exosomal protein, BPI, from SLE T cells. The exosomal BPI may contribute to SLE pathogenesis. BPI overexpression in T cells inhibited Treg population, leading to susceptible to inflammation responses. Lck-BPI Tg mice spontaneously developed autoimmune responses with early induction of serum IL-1β. Remarkably, T-cell-derived BPI-containing exosomes targeted several tissues (such as the liver, kidney, and joint) and also induced serum IL-1β in the recipient mice, leading to tissue inflammation. These data suggest that T-cell-derived BPI-containing exosomes may act as a causal factor of autoimmune responses.

To our knowledge, this report provides the first evidence that BPI overexpression attenuates Treg population and induces inflammation. Our scRNA-seq and T-cell differentiation analyses showed that BPI transgenic T cells displayed decreased Treg population; this may contribute to the induction of inflammatory responses in Lck-BPI Tg mice and SLE patients. Our scRNA-seq data also suggest that the decreased Treg population in Lck-BPI Tg mice may be due to upregulation of ZFP36L2, leading to Helios downregulation and subsequent Foxp3 downregulation in T cells. Besides suppression of Treg cells, BPI overexpression also induced inflammation. Consistently, BPI treatment *in vitro* enhances bacterial lipopeptides-induced secretion of multiple proinflammatory cytokines (TNF-α, IL-6, and IL-8) from human peripheral blood mononuclear cells (PBMCs) 39. Anti-BPI autoantibody levels are increased in patients with inflammatory diseases (such as systemic sclerosis and inflammatory bowel disease), whereas systemic sclerosis patients with high anti-BPI antibody levels show decreased skin inflammation in the absence of renal involvement 40. These reports are consistent with our finding that BPI overexpression contributes to inflammation and autoimmune diseases. Interestingly, T-cell-derived exosomes from Lck-BPI Tg mice induced IL-1β expression of wild-type recipient macrophages, suggesting that BPI overexpression in T cells contributes to inflammation at least partly through BPI-containing exosomes. Moreover, our scRNA-seq data showed an increased mRNA levels of S1PR1 in Lck-BPI transgenic T cells. S1PR1 signaling in T cells contributes to T-cell egress from lymph nodes to tissues 41, suggesting that BPI-S1PR1 signaling may facilitate migration of inflammatory T cells into inflamed tissues. Overexpression of S1PR1 in T cells from the brain tissue of multiple sclerosis patients is associated with neuroinflammation 42; therefore, upregulation of S1PR1 in T cells of Lck-BPI Tg mice may be also involved in the development of neuroinflammation. In addition, our scRNA-seq data revealed one interesting BPI-induced gene, ARCN1, which controls vesicle structure 43. BPI and ARCN1 proteins are also detected in exosomes from human urines and human thymic tissues, respectively 44, 45. The ARCN1 protein is mainly expressed in inflamed tissues 46. Another BPI-induced gene, VPS37b, is recently reported as a surface protein on the glioma-derived exosomes 47. It is possible that the BPI-ARCN1 axis or BPI-VPS37b axis in T cells facilitates exosome formation, leading to inflammatory exosome infiltration into target tissues. Collectively, BPI overexpression in T cells contributes to autoimmune responses through both intrinsic (inhibition of Treg population) and extrinsic (induction of inflammatory exosomes) pathways. Notably, distinguishing the specific role of BPI in T cells vs. that in exosomes is highly challenging if not impossible due to the lack of exosome-specific surface markers for depletion of exosomes *in vivo*. Nevertheless, it would be interesting to further understand the underlying mechanisms of BPI-induced inflammation via exosomes and Treg in the future.

SLE patients suffer from complex symptoms, including multi-tissue damages 1. The tissue tropism of SLE T-cell-derived exosomes may help understand the heterogeneous symptoms of SLE. In this study, we found that BPI-containing exosomes targeted the joint, liver, and kidney in mice, resulting in arthritis, hepatitis, and nephritis respectively. According to the clinical data of the 6 SLE patients enrolled in this study, all of the 6 SLE patients who had T-cell-derived, BPI-containing exosomes also developed arthritis. The data suggest that BPI-containing exosomes may deliver pathogenic factors into the joint and induce arthritis. For SLE nephritis, 2 of these 6 SLE patients also developed nephritis. SLE nephritis without the manifestation of proteinuria is difficult to be diagnosed at the early stage. Exosomes derived from Lck-BPI Tg T cells induce nephritis in all recipient mice, suggesting that BPI-containing exosomes may help early diagnosis of SLE nephritis. Although about half of SLE patients develop hepatitis, it is challenging to clarify whether hepatitis development in SLE patients is due to either SLE pathogenesis, infectious diseases, or therapeutic treatment 48. Notably, Lck-BPI Tg mice also spontaneously developed hepatitis, suggesting that hepatitis could be a consequence of the induction of exosomal BPI from SLE T cells. Taken together, BPI overexpression in T cells and T-cell-derived exosomes may be a potential biomarker for SLE arthritis, hepatitis, and nephritis.

## Supplementary Material

Supplementary figures.

## Figures and Tables

**Table 1:** Profile of SLE patients enrolled for exosome proteomics

SLE#	Gender	Age	SLEDAI	Duration (year)	dsDNA (U/mL)	C3 (mg/dL)	C4 (mg/dL)	WBC (/mm^3^)	PLT (1,000/mm^3^)	HgB (g/dL)	Arthritis	Nephritis	Hepatitis^§^	Cohort	

1	F	40	6	13	53.5	64.8	19.5	9250	402	12.1	1	1	1	1	
2	F	21	4	1	62.4	61.6	14.2	4210	317	12.8	1	0	0	1	
3	F	27	6	3	11.8	71.1	19.2	8600	252	12.9	1	0	0	1	
4	F	20	12	2.5	49.6	99.5	15.0	3670	205	12.2	1	1	0	1	
5	F	66	10	0.2	114.0	56.8	6.6	3400	203	10	1	0	1	2	
6	F	42	2	0.3	0.0	128.0	33.7	12,000	262	14.1	1	0	1	2	

Cohort #1, patients from the Division of Immunology and Rheumatology at Taichung Veterans General Hospital in Taiwan. Cohort #2, patients from the Division of Immunology and Rheumatology at Taipei Veterans General Hospital in Taiwan. §, patients with hepatitis during 2018-2020. F, female; SLEDAI, SLE disease activity index; C3, complement component C3; C4, complement component C4; WBC, white blood cell; PLT, platelet; HgB, haemoglobin.
