# Supplementary material for: BPI overexpression suppresses Treg differentiation and induces exosome-mediated inflammation in systemic lupus erythematosus
Source: Theranostics. 2021 Oct 25;11(20):9953–66. doi: 10.7150/thno.63743 (PMC8581436; doi:10.7150/thno.63743)
Supplement: Supplementary file 1 — Supplementary figures. [file thnov11p9953s1.pdf]

A

	SLE (n = 41)	HC (n = 40)
Gender (female, %)	38 (92.7)	34 (85)
Age at study entry	37.6 ± 13.7	39 ±16.2
SLEDAI	8.3 ± 6.9	NA
Disease duration (Years)	4.4 ± 4.5	NA
Anti-dsDNA antibody	94.9 ± 130.4	NA
C3 (mg/dl)	83.2 ± 28.4	NA
C4 (mg/dl)	16.2 ± 9.9	NA
WBC (/mm^3)	6,861 ± 2,625	NA
Platelet (1,000/mm^3)	240.4 ± 99.1	NA
HgB (g/dl)	11.7 ± 2.1	NA

B

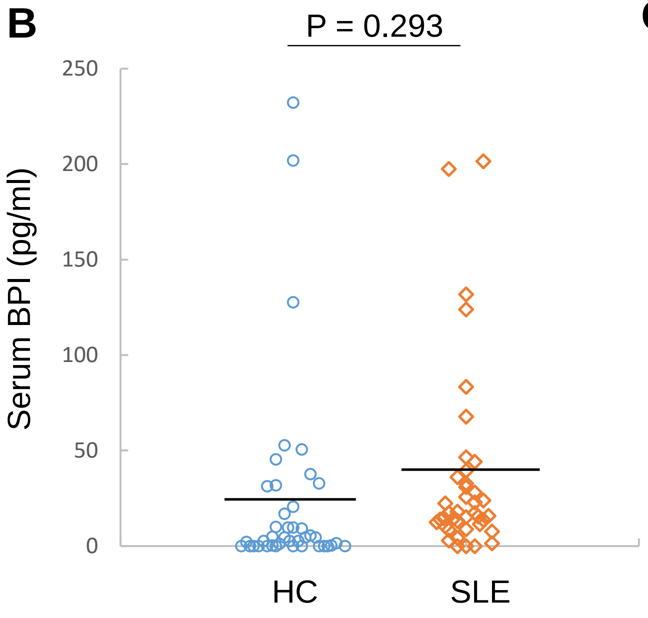

C

HC #1, T cells, BPI protein score: 0

HC #2, T cells, BPI protein score: 0

SLE #1, Peripheral blood T cells, BPI protein score: 130

1 M**RENMARGPC NAPRWASLMV LVAIGTAVTA AVNPGVVVR**I SQK**GLDYASQ**
51 **QGTAALQKEL KR**IKIPDYSD SFKIKHLGKG HYSFYSMDIR EFQLPSSQIS
101 MVPNVGLK**FS ISNANIK**ISG KWKAQKRFLK MSGNFDLSIE GMSISADLKL
151 GSNPTSGKPT ITCSSCSSHI NSVHVHISK**S KVGWLIQLFH K**KIESALRNK
201 MNSQVCEKVT NSVSSELQPY FQTLPVMTKI DSVAGINYGL VAPPATTAET
251 LDVQMKGEFY SENHHNPPPF APPVMEFPAA HDRMVYLGLS DYFFNTAGLV
301 YQEAGVLKMT LRDDMIPKES KFRLTTKFFG TFLPEVAKKF PNMKIQIHVS
351 ASTPPHLSVQ PTGLTFYPAV DVQAFAVLPN SSLASLFLIG MHTTGSMEVS
401 AESNR**LVGEL KLDRLLLELK** HSNIGPFPVE LLQDIMNYIV PILVLPRVNE
451 KLQKGFPLPT PAR**VQLYNVV LQPHQNFLLF GADVVYK**

SLE #2, Peripheral blood T cells, BPI protein score: 102

1 MRENMARGPC NAPR**WASLMV LVAIGTAVTA AVNPGVVVRI SQKGLDYASQ**
51 **QGTAALQKEL KR**IKIPDYSD SFKIKHLGKG HYSFYSMDIR EFQLPSSQIS
101 MVPNVGLK**FS ISNANIK**ISG KWKAQKRFLK MSGNFDLSIE GMSISADLKL
151 GSNPTSGKPT ITCSSCSSHI NSVHVHISK**S KVGWLIQLFH KK**IESALRNK
201 MNSQVCEKVT NSVSSELQPY FQTLPVMTKI DSVAGINYGL VAPPATTAET
251 LDVQMKGEFY SENHHNPPPF APPVMEFPAA HDRMVYLGLS DYFFNTAGLV
301 YQEAGVLKMT LRDDMIPKES KFRLTTKFFG TFLPEVAKKF PNMKIQIHVS
351 ASTPPHLSVQ PTGLTFYPAV DVQAFAVLPN SSLASLFLIG MHTTGSMEVS
401 AESNRLVGEL KLDRLLLELK HSNIGPFPVE LLQDIMNYIV PILVLPRVNE
451 KLQKGFPLPT PAR**VQLYNVV LQPHQNFLLF GADVVYK**

Supplementary Figure 1. BPI is induced in peripheral blood T cells of SLE patients, whereas soluble BPI is not significantly induced in the sera of SLE patients. (A) Profile of SLE patients (n = 41) and paired healthy control (n = 40). Data are presented as mean ± SD. (B) Soluble BPI levels in the sera from 41 SLE patients (including #1 ~ #6 used for exosome proteomics) and 40 healthy controls (HC) were measured by ELISA. (C) Identification of BPI by mass spectrometry-based protein sequencing of peripheral blood T cells from 2 SLE (#1 and #2) patients. Matched BPI peptides are shown in red. BPI was not detected in T cells from 2 healthy controls (HC, #1 and #2). Protein score is the sum of the highest ions score of MS/MS search for each distinct peptide.

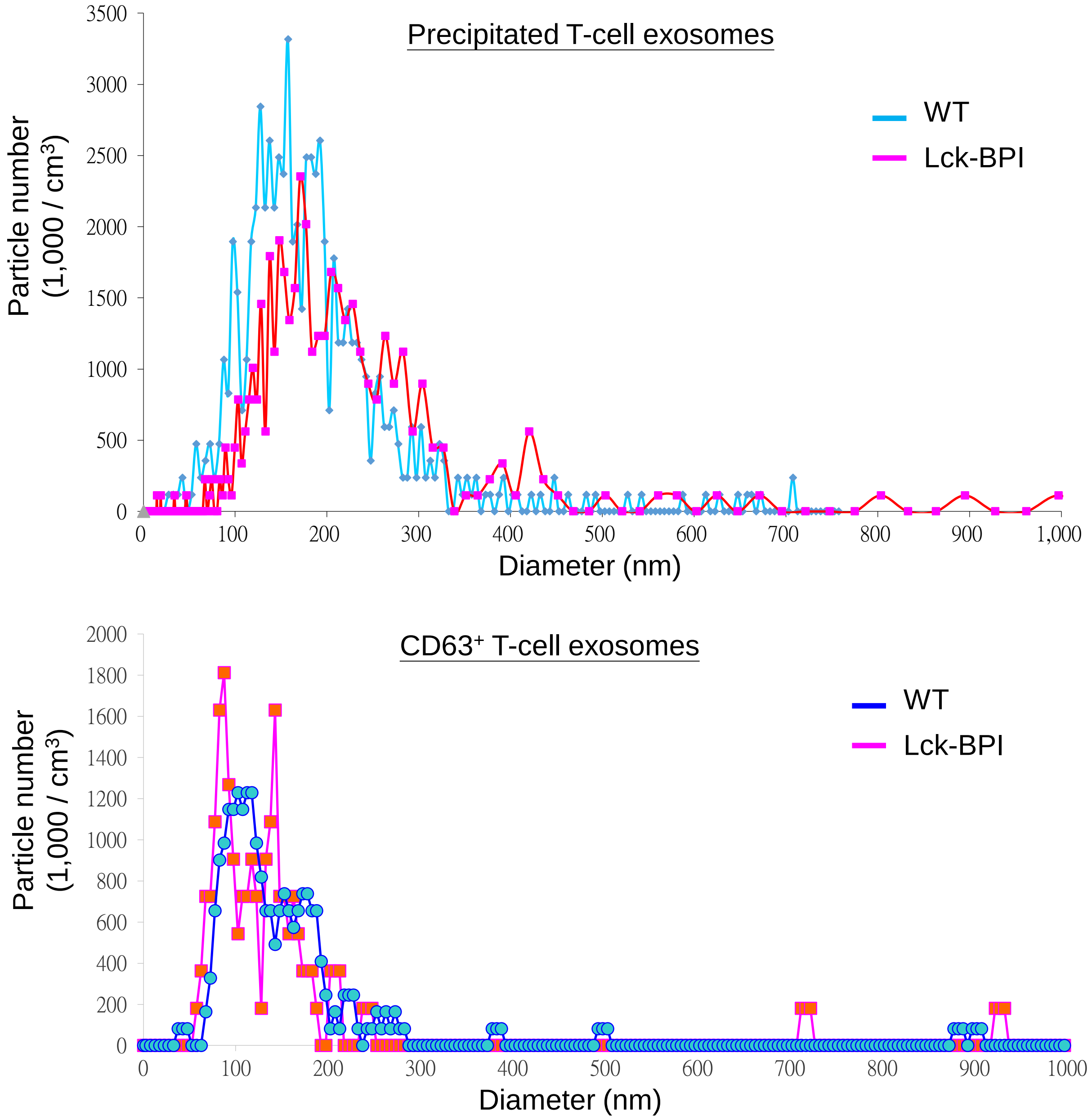

Supplementary Figure 2. The numbers and sizes of extracellular vesicles from Lck-BPI transgenic T cells are comparable to those of wild-type T cells. (**A**) ZetaView analysis of particle numbers and sizes of precipitated extracellular vesicles in supernatants from Lck-BPI Tg and wild-type (WT) T cells. Extracellular vesicles were precipitated by ExoQuick-TC. (**B**) ZetaView analysis of particle numbers and sizes of $CD63^+$ extracellular vesicles in supernatants from Lck-BPI Tg and wild-type (WT) T cells. Extracellular vesicles were precipitated by ExoQuick-TC, followed by resuspension. $CD63^+$ extracellular vesicles were further immunoprecipitated by anti-CD63 antibody-conjugated beads.

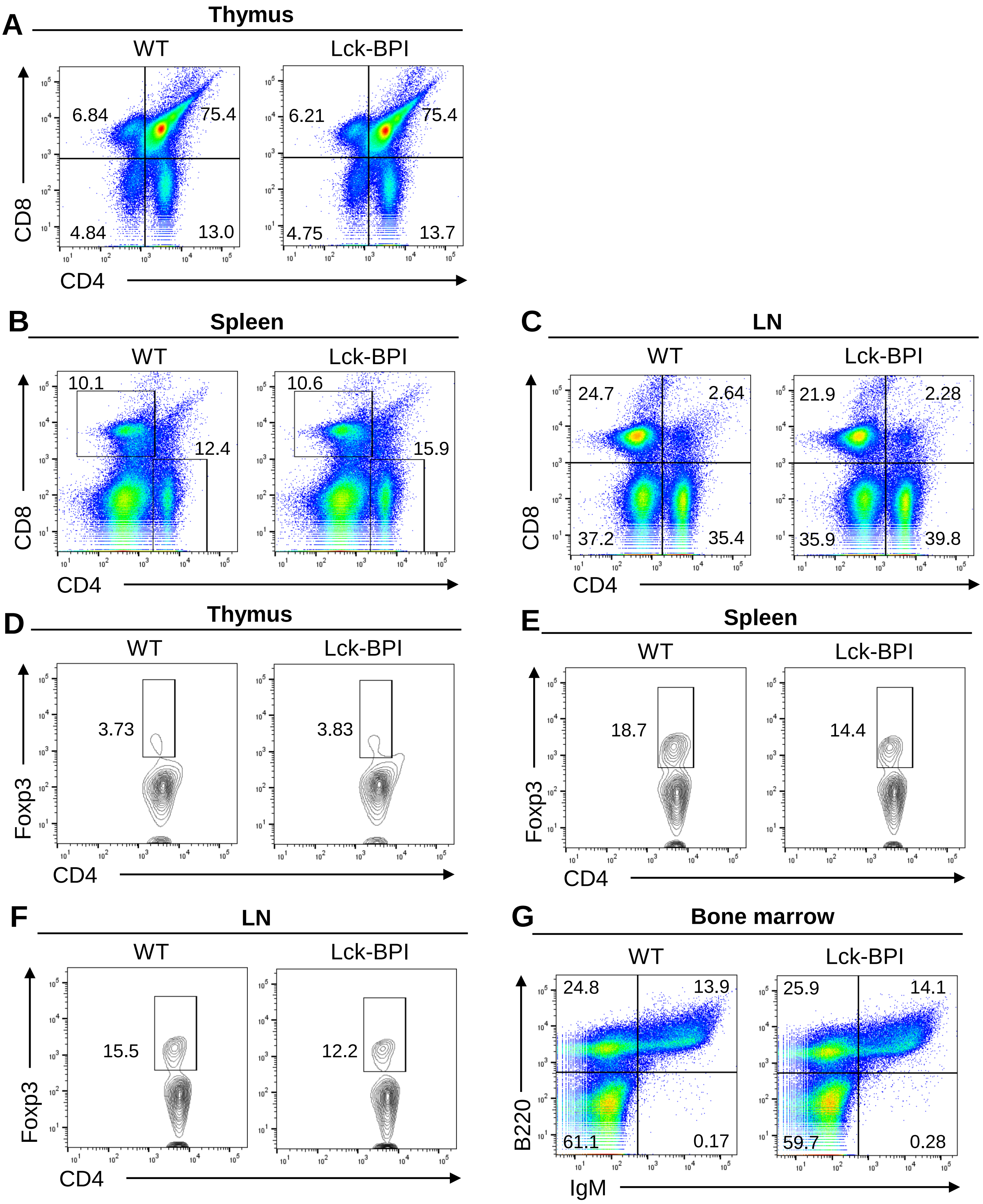

Supplementary Figure 3. Normal T-cell and B-cell development in thymus and bone marrow of Lck-BPI transgenic mice. (**A-E**) Flow cytometry analyses of T cells (A-C) and Treg cells (D-F) from the thymus, spleen, or lymph nodes of 5-week-old wild-type (WT) or Lck-BPI transgenic mice. (**G**) Flow cytometry analyses of B220$^+$ B cells from the bone marrow of WT or Lck-BPI transgenic mice. Data shown are representatives of three independent experiments.

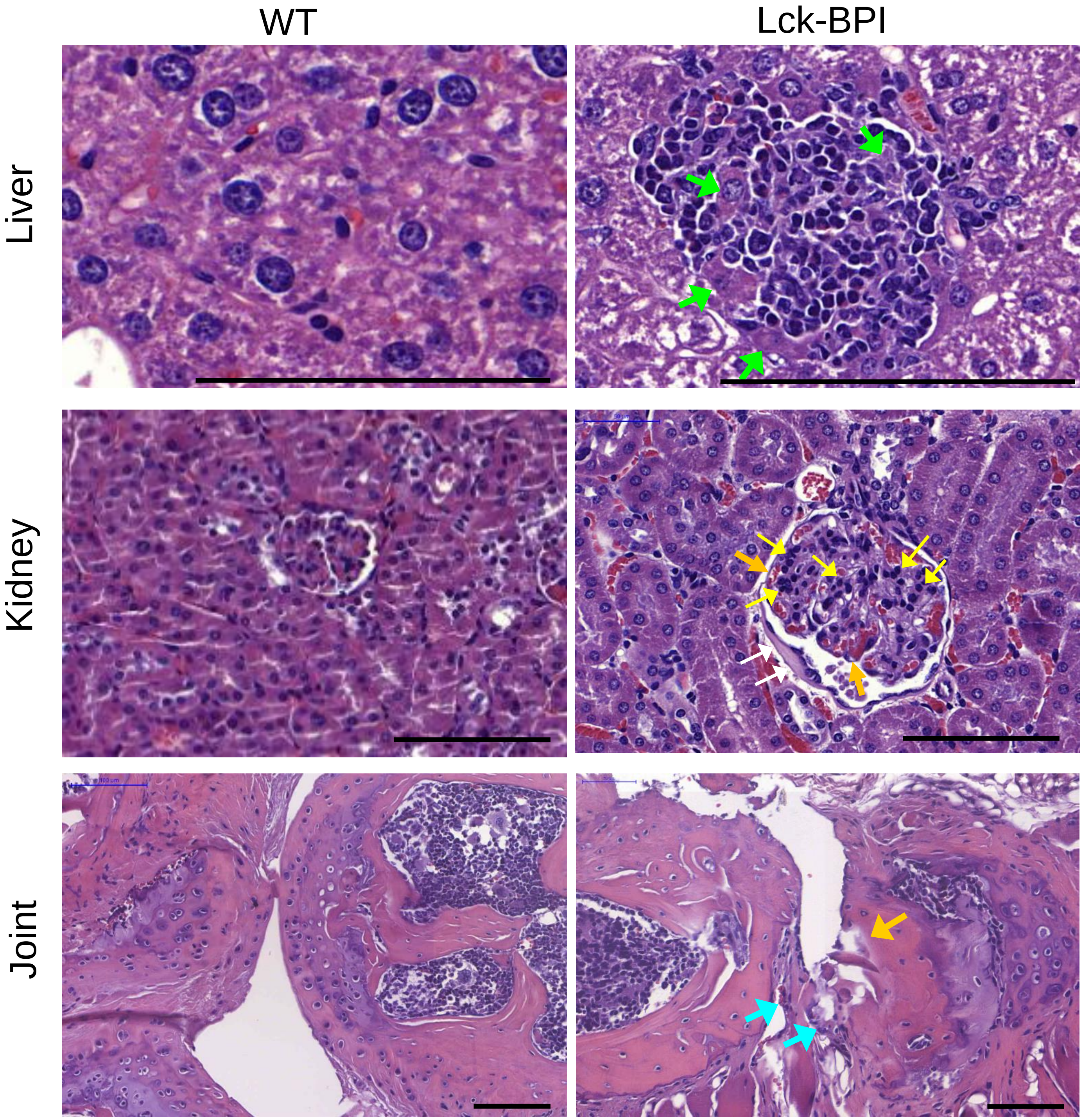

Supplementary Figure 4. Enlarged H&E staining images show hepatitis, nephritis, and arthritis in Lck-BPI transgenic mice. Hematoxylin and eosin (H&E)-stained sections of the liver, kidney, and joint from 8-month-old Lck- BPI Tg and WT mice. Scale bars, 100 μm. For the liver, green arrows denote hepatocytes with spotty necrosis in the inflammatory cell aggregation region of the liver tissue from Lck-BPI Tg mice. For the kidney, orange arrows denote thickening of glomerular basement membrane, white arrows denote thickening of Bowman's capsule, and yellow arrows denote mesangial expansion in the kidney of Lck-BPI Tg mice. For the joint, blue arrows denote leukocyte infiltration, and orange arrow denotes cartilage erosion in the joint of Lck-BPI Tg mice.

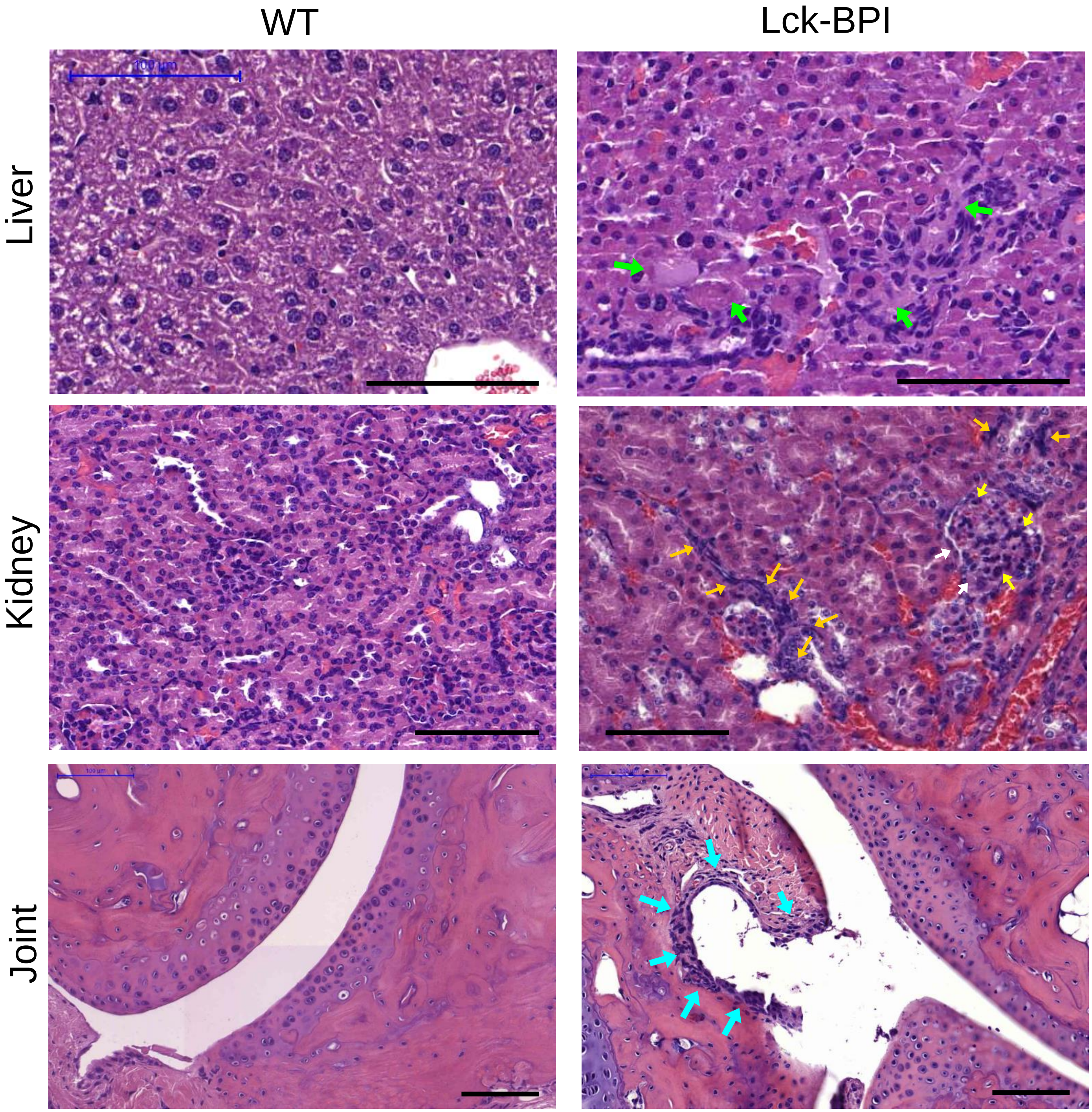

Supplementary Figure 5. Enlarged H&E staining images show hepatitis, nephritis, and arthritis in BPI-exosome recipient mice. Hematoxylin and eosin (H&E)-stained sections of the liver, kidney, and joint from WT-exosome or BPI-exosome recipient mice. Scale bars, 100 μm. For the liver, green arrows denote hepatocytes with spotty necrosis in the inflammatory cell aggregation region of the liver tissue from recipient mice. For the kidney, orange arrows denote immune cell infiltration, white arrows denote thickening of Bowman's capsule, and yellow arrows denote mesangial expansion in the kidney of recipient mice. For the joint, blue arrows denote leukocyte infiltration in the joint of recipient mice.

A

Downregulated Genes in Cluster 3	Fold (log2)	P value
CD8b-antibody seq	-3.094260431	0
Igkc	-1.621694935	4.36E-122
Hspa1b	-0.754517723	8.78E-73
Ighm	-1.2253256	1.14E-58
Hspa1a	-0.676283249	9.84E-57
Fos	-0.57525917	4.39E-52
Fosb	-0.523029493	4.94E-45
Eef1a1	-0.262209727	6.20E-40
Rps26	-0.314582233	9.48E-40
B2m	-0.323128326	1.36E-39
Rps5	-0.299563635	1.83E-37
Rpl17	-0.260282329	1.84E-35
Hspa8	-0.324023635	1.83E-33
Rps12	-0.314607672	3.89E-33
Zfp36	-0.457675745	9.71E-33
Rpl34	-0.269147633	6.87E-30
Hsp90ab1	-0.285256665	1.23E-29
Rpsa	-0.296276437	3.77E-29
Dusp2	-0.3568923	1.55E-28
Rps7	-0.263366033	1.97E-28
Ubb	-0.273926672	3.43E-27
Il2rb	-0.301019161	5.39E-27
Rpl37a	-0.26784481	1.22E-26
Cd8b1	-0.413206713	4.29E-26
Eef1b2	-0.289422956	4.62E-26
Rpl39	-0.253592355	5.88E-26
Rps15a	-0.268196341	6.30E-26
Rpl32	-0.277539907	6.88E-25
Rpl37	-0.264593473	1.95E-24
Rpl23a	-0.269575798	2.76E-24
Rpl13	-0.269317305	4.66E-24
Egr1	-0.387919942	5.84E-24
H2-K1	-0.25700867	1.28E-23

B

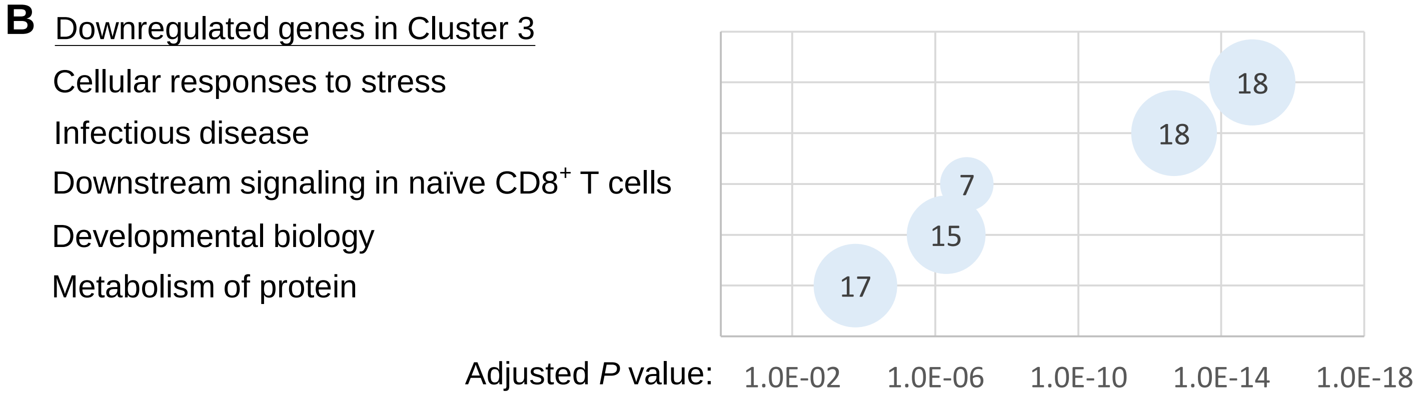

Supplementary Figure 6. The gene expression profile of the Cluster 3 cell population increased in Lck-BPI transgenic mice. (**A**) Downregulated genes in Cluster 3 compared to all other clusters. (**B**) KEGG pathway enrichment of downregulated genes in Cluster-3 T cells. Pathways belonging to different classifications are listed on the left of the plot. Varied numbers of genes enriched in individual pathways were presented by different diameter sizes and numbers for individual dots.

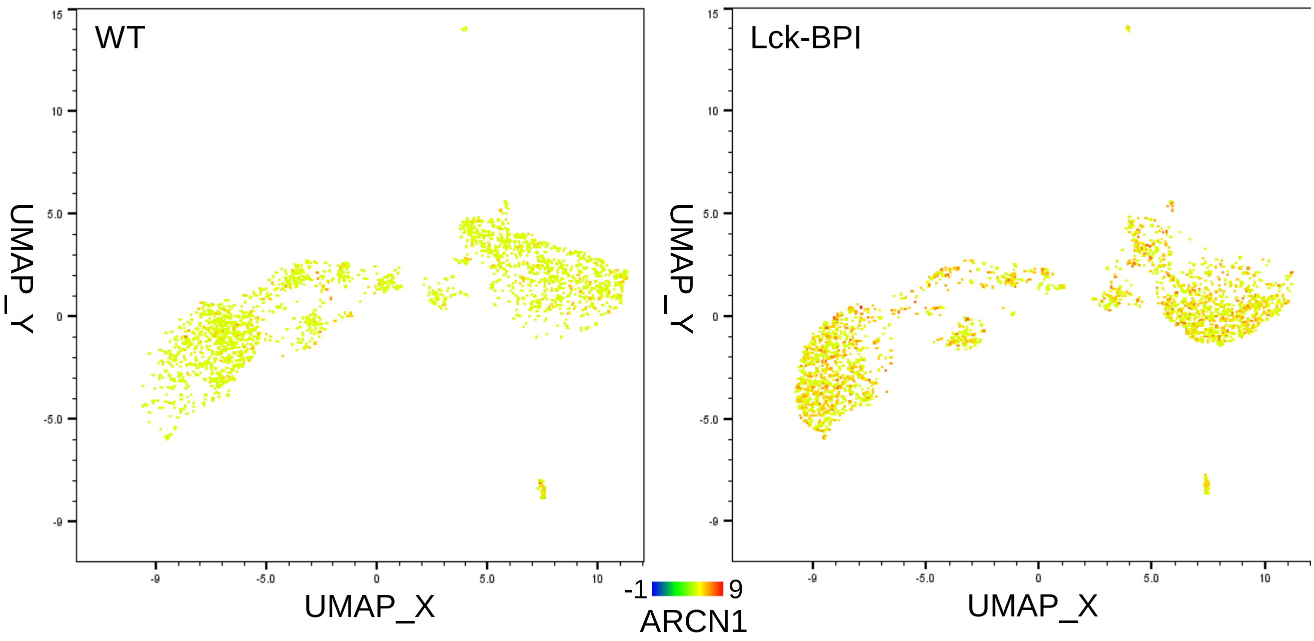

Supplementary Figure 7. ARCN1 levels are increased in all Clusters of Lck-BPI transgenic T cells.

Single-cell gene expression of ARCN1 in WT and Lck-BPI T cells.

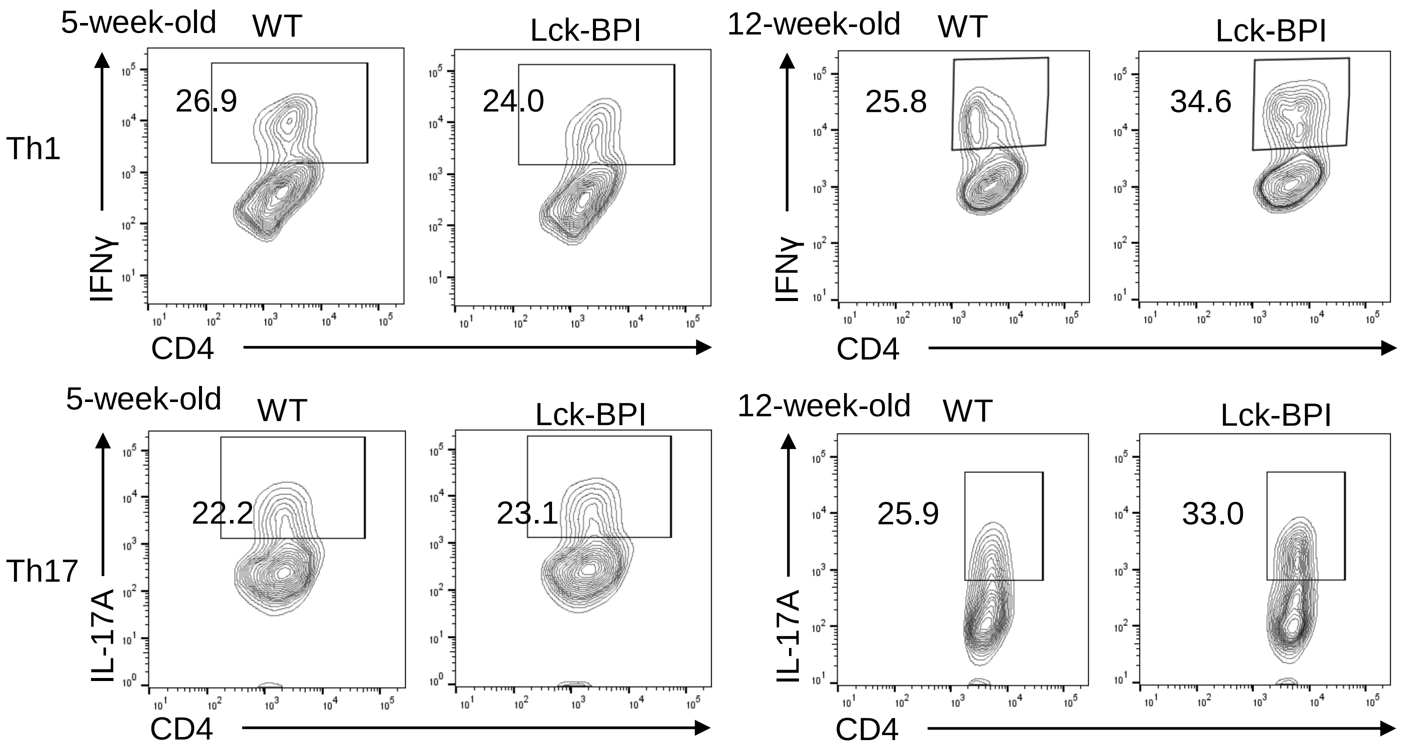

Supplementary Figure 8. Th1 and Th17 differentiation are enhanced Lck-BPI transgenic T cells. *In vitro* Th1 and Th17 differentiation of $CD4^+$ splenic T cells from 5-week-old (left panel) or 12-week-old (right panel) Lck-BPI Tg and WT mice. IFNγ-positive and IL-17A-positive $CD4^+$ T cells were analyzed by flow cytometry.
